# Exploring the diagnostic potential: magnetic particle imaging for brain diseases

**DOI:** 10.1186/s40779-025-00603-5

**Published:** 2025-04-27

**Authors:** Li-Shuang Guo, Yu An, Ze-Yu Zhang, Chen-Bin Ma, Jia-Qian Li, Zhen Dong, Jie Tian, Zhen-Yu Liu, Jian-Gang Liu

**Affiliations:** 1 School of Engineering Medicine, Beihang University, Beijing, 100191 China; 2 School of Biological Science and Medical Engineering, Beihang University, Beijing, 100191 China; 3 Key Laboratory of Big Data-Based Precision Medicine (Beihang University), Ministry of Industry and Information Technology of China, Beijing, 100191 China; 4 CAS Key Laboratory of Molecular Imaging, Institute of Automation, Beijing, 100191 China; 5 University of Chinese Academy of Sciences, Beijing, 100080 China

**Keywords:** Magnetic particle imaging, Brain diseases, Early diagnosis

## Abstract

Brain diseases are characterized by high incidence, disability, and mortality rates. Their elusive nature poses a significant challenge for early diagnosis. Magnetic particle imaging (MPI) is a novel imaging technique with high sensitivity, high temporal resolution, and no ionizing radiation. It relies on the nonlinear magnetization response of superparamagnetic iron oxide nanoparticles (SPIONs), allowing visualization of the spatial concentration distribution of SPIONs in biological tissues. MPI is expected to become a mainstream technology for the early diagnosis of brain diseases, such as cancerous, cerebrovascular, neurodegenerative, and inflammatory diseases. This review provides an overview of the principles of MPI, explores its potential applications in brain diseases, and discusses the prospects for the diagnosis and management of these diseases.

## Background

As a central component of the nervous system, the brain intricately regulates the pathophysiological processes in the human body. Its core is the neurovascular unit, which comprises neurons, microglia, astrocytes, vascular endothelial cells, and pericytes [1]. This complex network plays a pivotal role in governing cerebral blood flow and permeability of the blood–brain barrier (BBB), and maintaining the microenvironmental equilibrium [2]. Due to its complex structure, it is difficult for clinicians to quickly determine the cause of a disease based on clinical symptoms alone when the brain is subjected to local abnormalities or disorders. Brain diseases such as cancerous, cerebrovascular, and neurodegenerative diseases are responsible for a global health burden [3, 4]. Some of these diseases may not exhibit typical symptoms [5, 6], but are likely to cause irreversible damage by the time the symptoms manifest [6, 7], therefore leading to delays in the treatment. Thus, the early diagnosis of brain diseases is of significant importance for improving prognoses [4].

Most brain diseases show functional abnormalities before clinical symptoms manifest [8]. These abnormalities are usually discovered by measuring the electromagnetic fields generated by neuronal activity as well as hemodynamic or metabolic effects. For example, tumor-related metabolic molecules in cancerous diseases, such as acidic metabolic wastes, are products of metabolic reprogramming, and their abnormalities in concentrations often precede those in the tissue structure [9, 10]. These metabolic molecules have been used as markers for the early diagnosis of tumors [9]. Similarly, behavioral and cognitive abnormalities are hardly observable in the early stages of Alzheimer’s disease (AD). Potential biological biomarkers, such as low amyloid-b42 levels in the cerebrospinal fluid, have been proven to be useful for detecting brain abnormalities related to AD before brain damage and clinical manifestation [11, 12]. Additionally, synaptic dysfunction and abnormal neuronal signaling occur in the early stages of AD, before other pathological symptoms [13]. Hence, functional imaging technology plays a crucial role in the early detection and diagnosis of brain diseases. It potentially offers neuroscientists and neurosurgeons detailed insights into specific brain regions and functions, and the localization of affected areas in neurological diseases, facilitating uncovering of the fundamental cause of neurological symptoms [14].

Magnetic particle imaging (MPI) is a novel functional imaging technique that leverages the nonlinear response of superparamagnetic iron oxide nanoparticles (SPIONs) [15] and provides three-dimensional (3D) localization of lesions where the response of the SPIONs is abnormal. For MPI, a field-free region (FFR) is generated and driven to scan the entire field of view (FOV). The responding signal is acquired by inductive receive coils and further reconstructed into an image. Since the first system was developed in Germany in 2005 [16], MPI has been greatly developed with the continuous innovation of hardware systems and reconstruction algorithms. MPI offers the advantages of high sensitivity, high temporal resolution, and quantifiable measurements, making it an ideal choice for a variety of preclinical molecular imaging applications, such as cell tracing [17–21], angiography [22–25], and tumor imaging [26–29] and treatment [27].

Compared with traditional medical imaging methods such as positron emission tomography (PET), magnetic resonance imaging (MRI), and optical imaging, MPI has shown greater imaging advantage in the field of biomedicine. First, MPI offers high sensitivity and temporal resolution [30, 31], which enables the detection of very low concentrations of SPIONs and capturing the dynamic information about blood flow. This allows for the identification of weak signals at the early stages of disease or in very small areas of the brain. Second, compared to PET, the tracer SPIONs used in MPI not only do not have any ionizing radiation, but also have longer half-lives [32, 33]. This allows MPI to offer a safe and long-term effective monitoring process for patients. Third, MPI collects signals only from the biological tissues where SPIONs are aggregated, and therefore, there is no background interference in MPI images. Finally, in contrast to optical imaging, MPI has no depth limitation and therefore enables imaging of deep tissues for brain disease detection. Generally, MPI is well-suited as a powerful tool for detecting small lesions with weak signals which usually occur in the early stages of brain diseases, particularly for deep tissue lesion detection.

This review discusses the potential applications of MPI in brain diseases. It begins by outlining the basic principles of MPI, including imaging probes, hardware systems, and reconstruction algorithms. Then, we explore the preclinical applications of MPI in brain diseases. Finally, we discuss the potential directions for future research and development.

## Principle of MPI

**Fig. 1:**
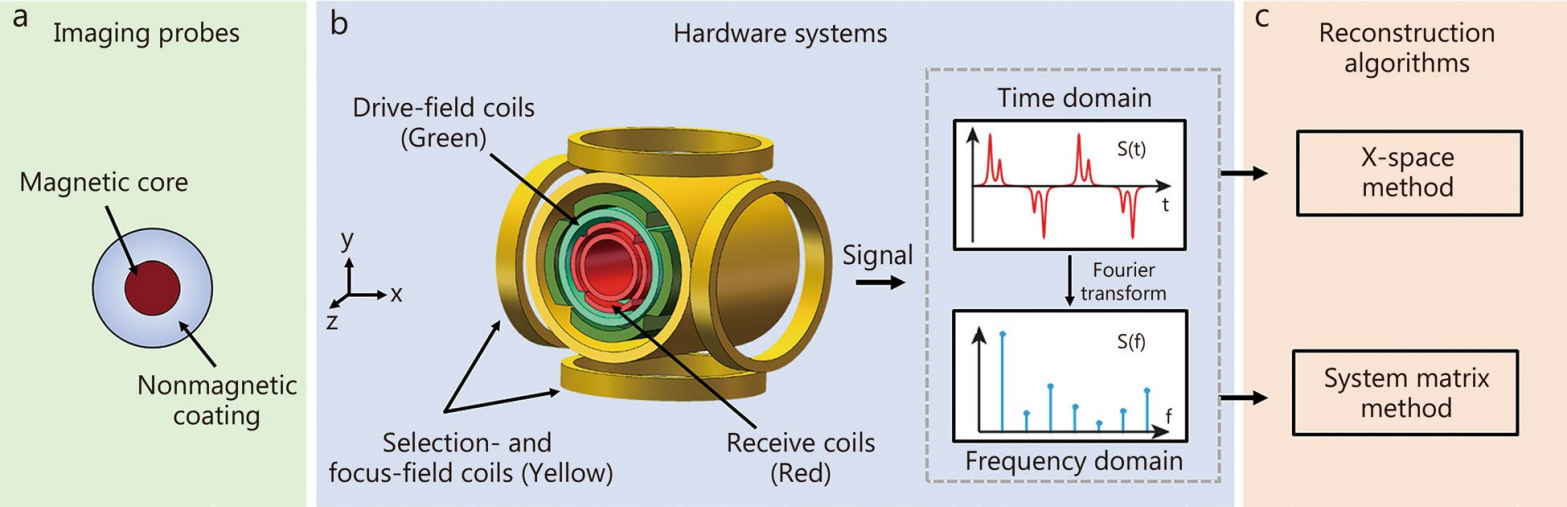
The component architecture diagram of magnetic particle imaging (MPI). Three key technologies for MPI are imaging probes (**a**), hardware systems (**b**), and reconstruction algorithms (**c**)

As shown in Fig. 1, high-quality MPI imaging relies on 3 key technologies: imaging probes (Fig. 1a), hardware systems (Fig. 1b), and reconstruction algorithms (Fig. 1c). These 3 key technologies will be described in further details.

### Imaging probes

As a type of probe, SPIONs consist of a magnetic core and a nonmagnetic coating (Fig. 1a). The former makes SPIONs magnetic, and the latter prevents them from agglomerating. Some types of SPIONs, like VivoTrax, Perimag, and Synomag-D, have been commercially applied in MPI. Furthermore, several types of SPIONs have been approved by the U.S. Food and Drug Administration for clinical applications, such as ferucarbotran (Resovist) and ferumoxytol (Feraheme). MPI employs SPIONs as tracers to directly measure the intensity and detect the spatial location of the SPIONs signal. Furthermore, the signal intensity of MPI only depends on the content of SPIONs in tissues, and there is a linear relationship between them, implying that MPI is a “hotspot” imaging technique without interference from background signal.

For MPI, the biocompatibility and safety of SPIONs are important issues to be considered when they are used for brain disease imaging. On the one hand, the core of SPIONs is composed of metallic oxides, commonly Fe_3_O_4_ and γ-Fe_2_O_3_, which exhibit good compatibility within biological systems [34] and therefore are often used as the material of the core in commercially available SPIONs. To further enhance the biocompatibility of SPIONs, the core is encased in a shell (i.e., the coating) with biocompatible materials, such as silicon dioxide. The surface of SPIONs can be modified to improve their stability in biological environments and prolong their circulation time in the bloodstream with common modification materials including polyethylene glycol, biopolymers (e.g., dextran, hyaluronic acid), and biocompatible polymers [34, 35]. The SPIONs with stable and long circulation facilitate the use of MPI for the long-term monitoring of brain diseases [34, 36], avoiding multiple injections [37]. Surface modifications can also introduce targeting ligands (e.g., antibodies, peptides, or small molecules) to increase the accumulation of SPIONs in specific organs or pathological areas, thereby enhancing imaging contrast. On the other hand, SPIONs can be metabolized through the reticuloendothelial system. This minimizes the risk of nephrogenic systemic fibrosis, which is a potential side effect associated with traditional contrast agents, particularly for patients with chronic kidney disease [38]. Some studies have suggested that the systemic administration of SPIONs does not result in their accumulation in the brain [39, 40], in contrast to gadolinium contrast agents which can deposit in the brain after repeated injections of gadolinium for MRI [41, 42]. Specifically, they can be phagocytosed and digested by macrophages or microglia, and then the degraded iron is incorporated into hemoglobin [43]. Nevertheless, it is still imperative to investigate whether SPIONs are completely cleared from the brain parenchyma or whether they increase neurotoxicity when the BBB is compromised [44]. Additionally, allergic reactions should also be considered for the applications of SPIONs, because a recent study reported allergic reactions to ferucarbotran in a small number of patients [45].

To enhance the imaging performance of SPIONs, researchers have fine-tuned their physical parameters, including size, shape, and composition of the core [34, 35]. Specifically, the core determines the magnetization response of SPIONs, and its size affects the magnetization strengths [34]. Larger particles have higher magnetization strengths and therefore higher signal-to-noise ratio (SNR). However, too large particles may cause a transition of particles magnetism from superparamagnetism to ferromagnetism, thereby degrading the MPI signal quality [46]. The shape of SPIONs affects their magnetic anisotropy, which in turn impacts the spatial resolution [34, 47]. Furthermore, compared to SPIONs with a core composed of metallic oxides, which often exhibit limited imaging performance, those with mixing metal alloys in their cores have been demonstrated to show enhanced magnetization response and superior imaging quality [34, 48, 49]. For example, Jiang et al. [49] designed a mixed metal–organic framework-derived carbon-supported ZnFe_2_O_4_/C, whose MPI signal intensity was 4.7-time higher than that of VivoTrax at an equivalent iron concentration, demonstrating excellent imaging performance in the ECA-109 tumor mouse models. The carbon-coated FeCo nanoparticles (FeCo@C) synthesized by Song et al. [48], with extremely high saturation magnetization, provide an MPI signal intensity that is 6-time and 15-time higher than the signals of VivoTrax and Feraheme, respectively. In this study, FeCo@C achieved high-performance breast cancer and brain tumor imaging and have the potential to be further utilized in photothermal and magnetic hyperthermia therapy.

In conclusion, the customization of SPIONs’ characteristics to cater to the nuances of specific diseases represents pivotal steps in facilitating the successful clinical adoption of MPI for brain imaging. Ongoing research endeavors are directed towards refining the design of SPIONs, promoting their penetration across the BBB and enhancing their imaging capabilities. These efforts facilitate unlocking the full diagnostic potential of SPION-based MPI in the context of detecting and characterizing brain diseases.

### Hardware systems

**Fig. 2:**
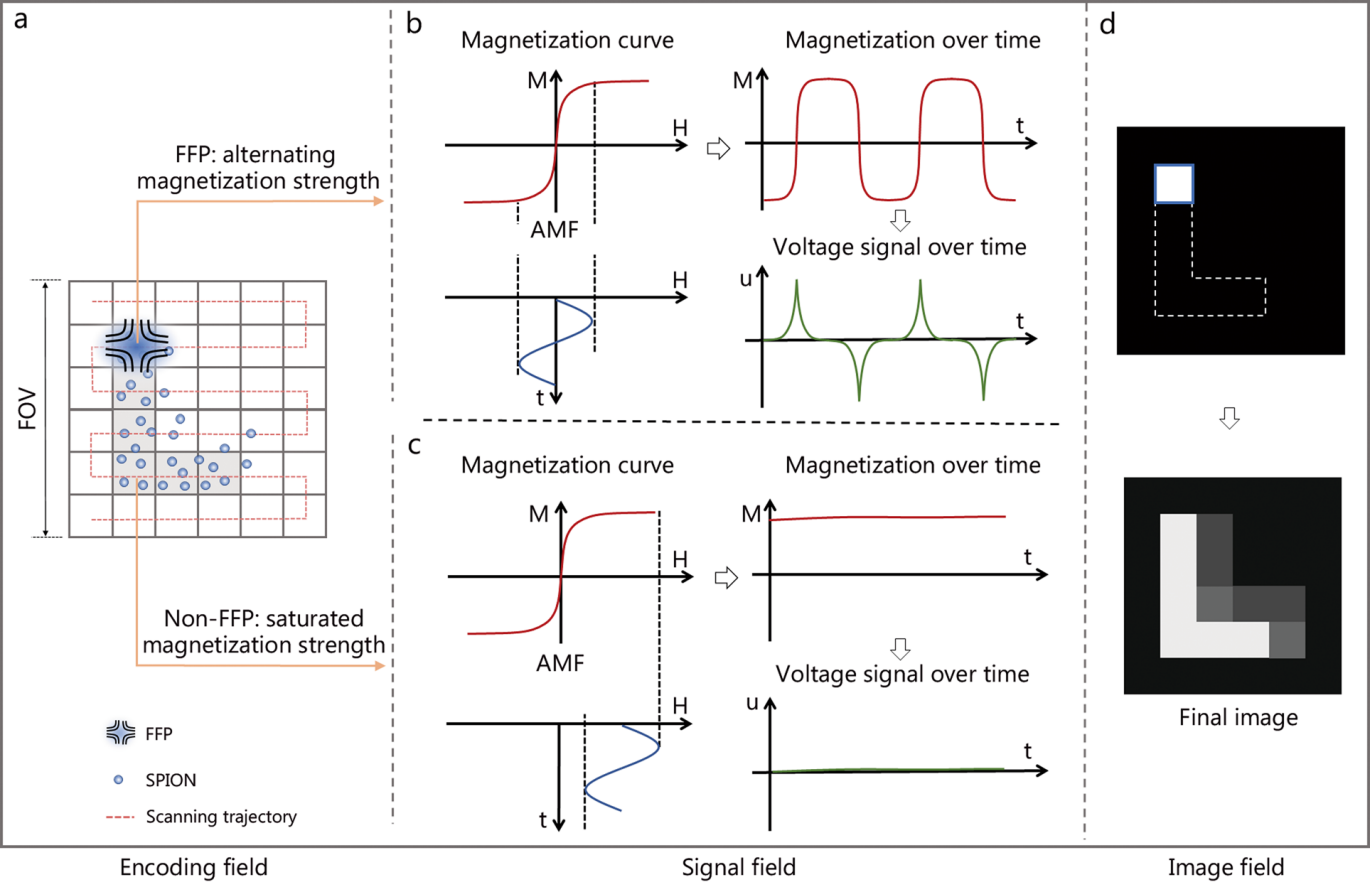
Fundamental principles of magnetic particle imaging (MPI) for field-free point (FFP). **a** FFP moves along a predefined trajectory in the imaging field of view (FOV). **b** When the FFP moves to a region containing superparamagnetic iron oxide nanoparticles (SPIONs), the SPIONs generate magnetization responses whose magnetization strength periodically changes under the drive of alternating magnetic field (AMF), and thereby producing alternating induced voltage in the receive coils. **c** In contrast, although driven by the same AMF as that for FFP, the SPIONs outside the FFP only generate a constant-like magnetization responses due to the magnetization saturation resulting from the selection field, and therefore producing no voltage in the receive coils. **d** After the FFP traverses the entire FOV, the spatial distribution of the magnetization response of SPIONs (i.e., the strength and location) is measured and recorded. After the image reconstruction, the final MPI image reflecting the spatial concentration distribution of the SPIONs is generated. M magnetization, H magnetic field, u voltage, t time

**Fig. 3:**
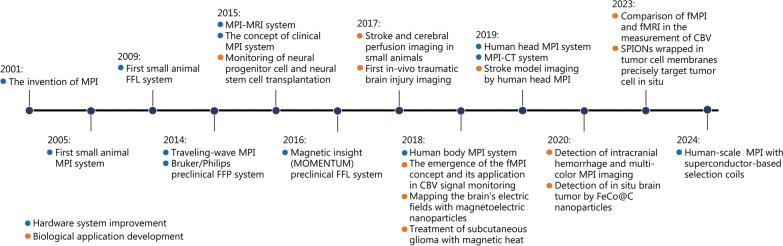
The development history of magnetic particle imaging (MPI) and its applications in brain diseases. After its invention in 2001, Gleich et al. [16] built the first small animal MPI system in 2005. Subsequently, several research groups conducted the exploration of MPI with different shapes [51, 52], and upgraded MPI to multimodal systems, such as MPI-magnetic resonance imaging (MRI) [53] and MPI-computed tomography (CT) systems [54]. Two commercial MPI systems were introduced in 2014 and 2016, subsequently experiencing widespread adoption. At the same time, MPI began to be applied to neuroimaging [33], where its efficacy was investigated across various diseases. With the emergence of human-sized systems [55, 56], the application scenario has been further extended to the simulation of brain diseases in human models [56]. In 2018, the concept of functional magnetic particle imaging (fMPI) was proposed [57], effectively promoting the exploration of MPI in the field of brain function [58, 59]. After 2018, the application of MPI in brain diseases has been expanded, including brain tumors [48, 65, 66], cerebrovascular diseases [60, 61, 64], and neurodegenerative diseases [62]. In 2024, the first human-scale MPI system with superconductor-based selection coils was developed [63]. FFL field-free line, FFP field-free point, CBV cerebral blood volume, SPIONs superparamagnetic iron oxide nanoparticles

As shown in Fig. 1b, a typical MPI system comprises three key components (i.e., selection- and focus-field coils, drive-field coils, and receive coils), which generate selection/focus field, alternating magnetic field (AMF), and induced voltage, respectively. The selection field saturates the magnetization of SPIONs in the magnetic field except for FFR wherein SPIONs generate magnetization response according to steady-state Langevin model [50]. FFR can be a field-free point (FFP) or a field-free line (FFL). There is only one FFR in the selection field which can move along a predefined trajectory under the drive of the focus field (Fig. 2a). When the FFP moves to a region containing SPIONs, the SPIONs generate magnetization responses whose magnetization strength periodically changes under the drive of AMF, and thereby generating alternating induced voltage in the receive coils (Fig. 2b). In contrast, although driven by the same AMF as that for FFR, the SPIONs outside the FFR only generate a constant-like magnetization response due to the magnetization saturation resulting from the selection field, and therefore producing no voltage in the receive coils (Fig. 2c). Thus, after FFR traverses the entire FOV, the spatial distribution of the magnetization response of SPIONs (i.e., the strength and location) is measured and recorded (Fig. 2d). Due to technological progress, a variety of MPI systems have emerged [51, 52]. Figure 3 [16, 33, 48, 51–66] provides a historical overview of MPI, highlighting the advancements in hardware systems and the expansion into diverse biological applications.

**Fig. 4:**
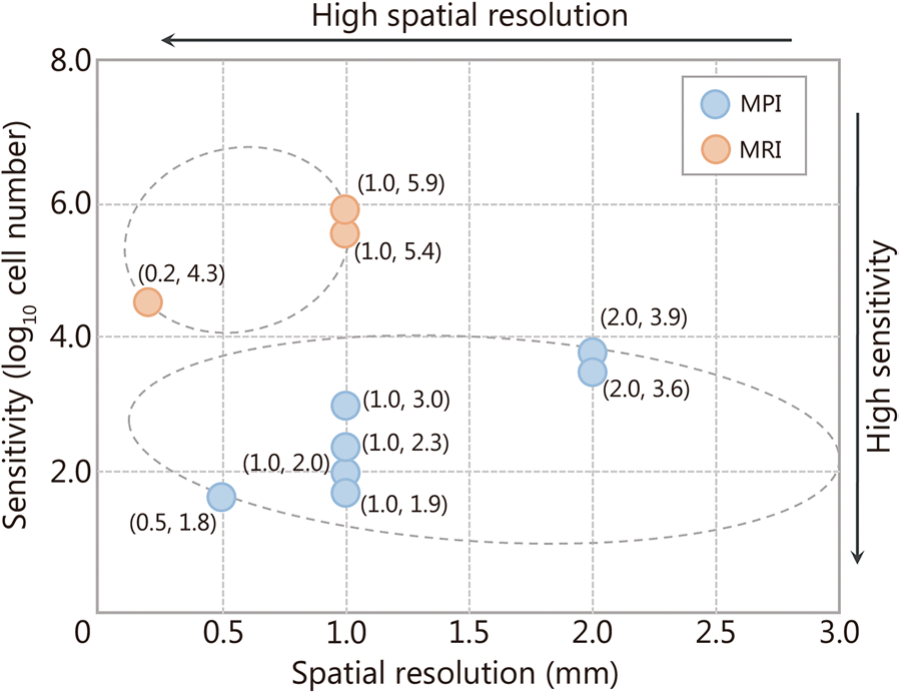
Visual comparison of sensitivity and spatial resolution between magnetic particle imaging (MPI) and magnetic resonance imaging (MRI). The circles represent the sensitivity (vertical axis) and spatial resolution (horizontal axis), which were measured by the same MPI (blue) or the same MRI (orange). Here, the sensitivity is defined as the logarithm of the number of detectable cells. The sensitivity of MPI is within 10,000 cells [20, 30, 33, 66, 69, 70] with the highest sensitivity approximating 69 mesenchymal stem cells [68]. The sensitivities of MPI are higher than those of MRI. However, MPI slightly falls short of MRI in spatial resolution [30, 60, 68]. The metrics of MPI and MRI were measured at the small animal aperture

The performance of MPI is heavily reliant on the hardware design, which is evaluated with temporal resolution, sensitivity, and spatial resolution as the key metrics. A well-designed MPI system can visualize biological processes in vivo with a high temporal resolution, nanomolar sensitivity, and millimeter spatial resolution [32, 67]. Table 1 [20, 30, 60, 62, 68] provides a quantitative comparison of these 3 metrics between MPI and MRI in specific applications, and Fig. 4 [20, 30, 33, 60, 66, 68–70] visually contrasts their sensitivity and spatial resolution.

**Table 1:** Quantitative comparison of three metrics between magnetic particle imaging (MPI) and magnetic resonance imaging (MRI) in specific application scenarios

Scenarios	Methods	Gradient (T/m)	Sensitivity	Temporal resolution	Spatial resolution	Findings	References
Detection of MSCs	MPI	3	4000 cells (76 ng Fe)	–	2 mm	The high sensitivity of MPI facilitates tracking cells and detecting tumor metastasis	[30]
	MRI	3	256,000 cells (9.01 × 10^16 19^F)	–	1 mm		
CAR T-cell tracking	MPI	6	2000 cells	–	1 mm	MPI provides a new way for immunization therapy with high sensitivity	[20]
	MRI	7	20,000 cells	–	200 μm		
Ischemic stroke	MPI	2.5	–	21.5 ms	3 mm	MPI with high temporal resolution is helpful to monitor acute vascular diseases	[60]
	MRI	7	–	177 ms	130 μm		
Brain perfusion	MPI	2.5	About 69 MSCs (896 pg Fe)	21.5 ms	500 μm	MPI can detect a few tracers with high temporal resolution and has potential for early detection of cerebrovascular diseases	[68]
	MRI	7	–	177 ms	130 μm		
Measurement of CBV	fMPI	2.83	CNR = 12–29	–	3 mm	fMPI with a low gradient provides much higher CNR than fMRI, promoting its potential to detect weak signals of diseases	[62]
	fMRI	9.4	CNR = 5.95	–	375 μm (smooth to 3 mm for comparing)		

*fMPI* functional magnetic particle imaging, *fMRI* functional magnetic resonance imaging, *CNR* contrast-to-noise ratio, *MSCs* mesenchymal stem cells, *CAR* chimeric antigen receptor, *CBV* cerebral blood volume

At present, for MPI, over 2000 frames per second (FPS) have been achieved in monitoring blood-flow dynamics [31], and 46 FPS have been achieved in stroke imaging [60], demonstrating evident advantages of MPI over MRI in temporal resolution. A preliminary human-sized MPI system has also achieved a temporal resolution of 2 FPS in brain applications [56]. Recently, the human-scale MPI system with a superconductor aperture was reported [63], but the 4 FPS did not meet expectations [67]. However, high-speed imaging in human-scale MPI systems is not an unattainable goal, because the imaging speed primarily relies on the chosen excitation frequency and trajectory density rather than the aperture size [71].

Sensitivity, defined as the minimum detectable amount of magnetic material, is another significant advantage of MPI. An in vitro study has demonstrated that MPI can achieve the detection of 200 labeled cells [33], with the potential for further enhancement. This sensitivity surpasses that of other imaging modalities. It is noteworthy that Graeser et al. [23, 68] have introduced an innovative method to improve the sensitivity of MPI. They designed a murine-specific surface coil that detected perfusion in the small arterial vessels within the brain and retina of a living mouse model. This advancement led to a remarkable sensitivity threshold of 896 pg Fe, equal to the detection of approximately 69 stem cells, based on the assumption that each cell contains 13 pg Fe [68]. The researchers predicted that the sensitivity of MPI could be improved to fewer than 10 cells [68, 69], marking an improvement of 3 orders of magnitude over the sensitivity of MRI. These advancements have laid the groundwork for significantly heightening sensitivity in human-scale MPI, rendering MPI an increasingly attractive imaging modality for a broad spectrum of applications, particularly in the context of brain disease diagnostics.

The fundamental spatial resolution of MPI depends on the saturation magnetization property of the SPIONs and the gradient strength of the selection field [72]. Most MPI systems with a 6.3 T/m gradient strength have a spatial resolution within the range of 1–2 mm [25]. To boost the spatial resolution of MPI, researchers have proposed a traveling wave MPI, which could provide a high magnetic field gradient, enabling a spatial resolution of 330 μm while retaining a mouse-sized FOV [52]. On the other hand, novel tracers such as super ferromagnetic iron oxide nanoparticles have achieved an ideal step-like magnetization response to improve image resolution to 0.15 mm in phantoms [73]. The improvement and combination of magnetic field gradients and novel tracers will be expected to improve the spatial resolution of MPI in brain imaging.

These 3 metrics are interdependent and profoundly influence the intricacy of MPI hardware systems. The diverse scenarios in medical applications require different settings and combination of these metrics, and therefore, specialized MPI systems must be designed to meet the needs of multiple applications. In the context of brain tumor imaging, there is a premium on high sensitivity and high spatial resolution to detect residual tumor tissues, which often have relatively low tracer accumulation. High spatial resolution is particularly important for precisely delineating the margins of tumors. In contrast, the systems for cerebrovascular diseases need high temporal resolution to swiftly collect dynamic changes in intravascular magnetic particles, detect the lesion site within a safe period, and take prompt intervention measures. Neurological applications require maximal sensitivity to capture weak electrical signals. A “one-size-fits-all” system is necessary and needs to balance all metrics. Such a system is beneficial for practical applications, as it allows physicians to consider various imaging needs associated with diverse diseases.

### Reconstruction algorithms

The primary objective of MPI reconstruction is to use the induced voltage signal (recorded by a receive coil) to characterize and visualize the spatial concentration distribution of SPIONs. Two methods are commonly employed for MPI image reconstruction: system matrix and X-space methods (Fig. 1c).

System matrix represents the relationship between the spatial concentration distribution of the SPIONs and the measured signal of the receive coil in the frequency domain. It can be constructed either by mathematical modeling or by measuring samples containing the solution of SPIONs and moving along a specific trajectory in FOV. The system matrix has to be calibrated before its reconstruction. The calibration process is very time-consuming due to the large size and high memory requirements of system matrix [74]. Additionally, the accuracy of the reconstruction depends on the behavior of SPIONs in tissue, which differs significantly from their behavior in solution, leading to a bias in obtaining the system matrix.

In contrast, X-space method can directly reconstruct MPI images from time-domain signals, and the entire process is fast and robust without the need for complex calibration and iterative process [50, 75]. This method reconstructs the spatial concentration distribution of SPIONs using velocity compensation, spatial discretization, and coordinate mapping. Compared to system matrix reconstruction, X-space is computationally simpler and faster, and is suitable for the application scenarios that require high imaging speeds [50]. Most commercial systems adopt X-space method for image reconstruction, such as MOMENTUM system and Berkeley system.

Additionally, deep learning technology has emerged to facilitate high-performance MPI in many ways [76], such as system matrix calibration [77–79], image resolution improvement [80–82], and quantitative prediction [83]. These methods help address the shortcomings of traditional reconstruction methods, and their combination further accelerates the development of MPI into clinical applications.

## Exploration of MPI in diagnosis of brain diseases

To the best of our knowledge, the applications of MPI in brain imaging began with the observation and transplantation of neural progenitor cells (NPCs) in 2015 [33]. NPCs, the offspring of stem cells, are programmed to differentiate into specific types of nerve cells. NPC transplantation has emerged as a promising therapeutic approach for a range of neurodegenerative disorders, including AD, Parkinson’s disease (PD), and Huntington’s disease (HD) [84–86]. In a pivotal in vivo study, SPION-labeled NPCs were implanted into the forebrains of immunosuppressed rats, and then their developmental trajectory was subsequently monitored using MPI [33]. This groundbreaking research marks the pioneering use of MPI for cell tracking, thereby establishing a solid foundation for further investigation of MPI’s potential in the diagnosis of brain diseases. The potential of MPI in the diagnosis of brain diseases, ranging from cancerous and cerebrovascular to neurodegenerative, as well as inflammatory diseases, is a subject of ongoing exploration. Table 2 [26, 33, 48, 56, 57, 60–62, 66, 68, 70, 87, 88] encapsulates some representative studies that have investigated the application of MPI in the detection of these brain diseases.

**Table 2:** Summary of recent studies investigating the use of magnetic particle imaging (MPI) for imaging and detecting brain diseases

Imaged models	Tracers	Scanner (mode^*^)	Findings	References
Xenograft tumor-in-situ glioma mice	Multimodality nanoparticles	MOMENTUM preclinical scanner (FFP, X-space)	MPI signals in brain tissue can increase 17.1 times after injection and MPI signals can be observed with rich blood supply	[26]
Xenograft tumor-in-situ glioma mice	FeCo@C	MOMENTUM preclinical scanner (FFP, X-space)	FeCo@C provides an MPI signal intensity that is 6-time and 15-time higher than the signals from VivoTrax and Feraheme, respectively	[48]
Xenograft tumor-in-situ glioma mice	Nanoparticles-covered the membrane of glioblastoma cells	MOMENTUM preclinical scanner (FFP, X-space)	MPI signal does not decay with tissue depth and shows excellent sensitivity for thousands of cells	[66]
Brain cancer xenografts	Lactoferrin-functionalized nanoparticles	MOMENTUM preclinical scanner (FFP, X-space)	The agent can detect 1.1 ng of iron (SNR was about 3.9). Lactoferrin coupling and external magnet can improve the tumor localization	[70]
C6 brain glioma cells in vitro	Lactoferrin-functionalized nanoparticles	Custom-built magnetic particle spectrometer	Nanoparticles with Lactoferrin have increased 5 times signal intensity compared to non-targeted particles	[87]
Neural progenitor cells in rat	Resovist	Custom-built MPI systems (FFL, X-space)	The detection limit is 200 cells (5.4 ng Fe) in vitro, and in vivo monitoring of human neural graft clearance is over 87 d in rat brain	[33]
Cerebral perfusion in healthy mice	Perimag	Dedicated surface coil for mice (FFP, system matrix)	MPI can detect tracer samples containing only 896 pg iron (about 69 cells), and even small vessels (150 µm diameter) and anatomical structures	[68]
Ischemic stroke model in mice	LS-008	Bruker preclinical System (FFP, system matrix)	MPI can be used for real time detection of perfusion deficits associated with ischemic stroke	[60]
Intracranial hemorrhage model in mice	Perimag and Synomag-D	Bruker preclinical system (FFP, system matrix)	Multi-contrast MPI can differentiate clotted blood from active bleeding. The bleed can be detected in 3 min, and the quantitative range is 0.003 – 0.06 μl/s	[61]
Healthy rhesus macaque	Mag3200	Hand-held MPI detector	The detector has a detection limit of about 125 ng Fe and can in vivo measure cerebral particle concentration changes	[88]
Stroke phantom	Perimag	Novel MPI head scanner (FFP, system matrix)	A system can achieve a sensitivity limit of 14.7 ng Fe/ml at a frame rate of 2 Hz and a spatial resolution of 5 mm	[56]
Hypercapnic model in rat	SPIONs with a 25 nm core and a polyethylene glycol carboxyl coating	Single-sided detector	MPI could measure CBV changes during hypercapnia with a CNR of 50	[57]
Hypercapnia model in rat	Synomag-D	Home-built rodent scanner (FFL, system matrix)	The average CNR of CBV in fMPI was approximately 2 – 6 times higher than that in fMRI	[62]

^*^Mode includes scanning mode (FFP or FFL) and image reconstruction method (system matrix or X-space reconstruction methods) of the scanner

*FFL* field-free line, *FFP* field-free point, *SNR* signal-to-noise ratio, *CNR* contrast-to-noise ratio, *fMPI* functional magnetic particle imaging, *fMRI* functional magnetic resonance imaging, *CBV* cerebral blood volum

### Cancerous diseases

The initial focus of MPI research within the field of oncology is to use SPIONs to monitor tumorigenesis, evaluate tumor progression, and assess the tumor microenvironment, which plays a significant role in the diagnosis and therapeutic management of various malignancies. Brain tumors represent a grave threat to human health, with their invasive nature often leading to rapid neurological deterioration and a poor prognosis [89, 90]. The presence of the BBB and the blood–brain tumor barrier poses a challenge for targeting brain tumors with conventional methods [91].

**Fig. 5:**
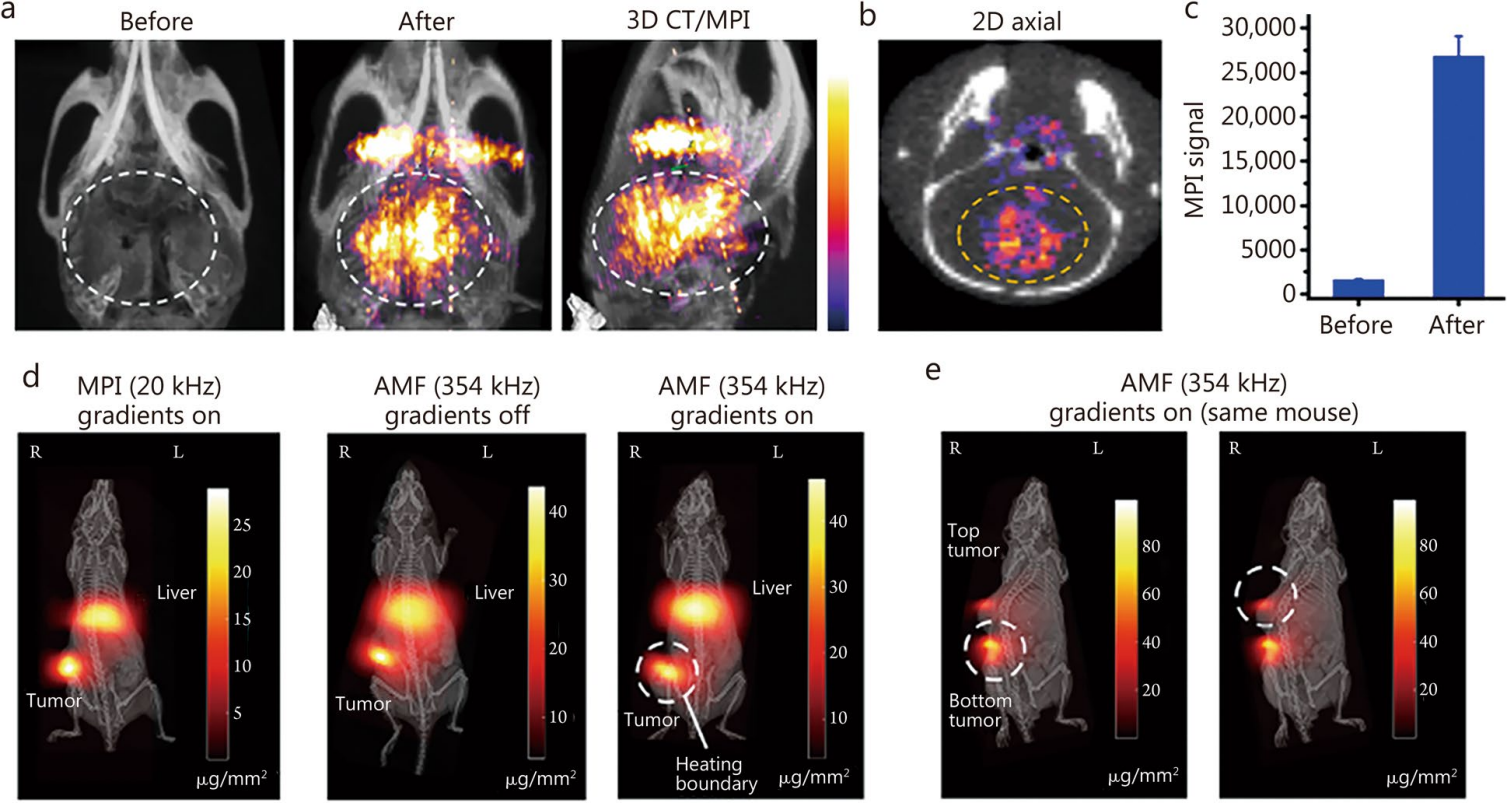
Magnetic particle imaging (MPI) detection and image-guided magnetothermal therapy for brain tumors. Imaging of orthotopic brain tumor xenografts in mice with multimodality nanoparticles (**a**–**c**). In the three-dimension (3D) computerized tomography (CT)/MPI images (**a**) and two-dimension (2D) axial image (**b**) of the mouse head, the brain areas showed enhanced MPI contrast after injection compared with that of pre-injection (**c**). Reproduced with permission from Ref. [26] (**a**–**c**). **d** MPI used SPIONs and an alternating magnetic field (AMF) to achieve magnetic hyperthermia for subcutaneous tumors. The heating site was controlled by moving the field-free line (FFL) to avoid thermal damage to the liver. **e** The top and bottom tumors in a mouse could be heated separately by moving the FFL. Reproduced with permission from Ref. [65] (**d** and **e**)

MPI, combined with tumor-specific SPIONs, offers a novel method for visualizing precise and real-time 3D images of tumor distribution. SPIONs can passively diffuse through leaky vessels and subsequently accumulate in the tumor due to the enhanced permeability and retention effect, exhibiting an excellent contrast at brain tumor sites. Yu et al. [32] demonstrated that the intravenous administration of commercial SPIONs such as LS-008 to tumor-bearing mice resulted in discernible tracer accumulation in the tumor with minimal background interference for up to 6 h post-injection. Multimodal SPIONs, like the multimodality—MPI, MRI, photoacoustic, fluorescent—nanoparticles [26], have also showed higher contrast signals in MPI (Fig. 5a–c) [26] in xenograft tumor-in-situ glioma mice compared to other modalities. Furthermore, targeted aggregation of SPIONs within tumors can be enhanced by active conjugation with specific ligands or molecular targets. For instance, lactoferrin-functionalized SPIONs have demonstrated a marked increase in cellular internalization after a 24-h co-incubation with C6 glioma cells, resulting in a 5-time enhancement in signal intensity compared to non-targeted particles [87]. This targeted approach highlights the potential of MPI for glioma-specific diagnostics. Innovative strategies, such as the encapsulation of magnetic nanoparticles within biomimetic tumor cell membranes, have been employed to achieve targeted localization of orthotopic gliomas in murine models [66]. This approach harnesses the inherent targeting capabilities of the tumor cell membrane to facilitate the delivery of diagnostic probes directly to the tumor site. Additionally, external magnetic force has been shown to drive the transmigration of SPIONs across the BBB [92], offering an alternative mechanism for achieving SPIONs accumulation within tumor cells [70]. In the context of cancer metastasis, the high sensitivity of MPI is particularly valuable for detecting the spread of cancer cells to the brain [93, 94]. Study by Melo et al. [95] has demonstrated the potential of MPI in tracking cancer cell metastasis in murine models, underscoring its potential for clinical application in the detection of brain tumor metastases. The application of MPI extends beyond diagnostics, holding promise in the field of therapeutics [96]. The combination of SPIONs with AMF can induce localized hyperthermia at the site of nanoparticle accumulation by controlling FFL (Fig. 5d, e) [65], which has shown clinical promise in the treatment of glioblastoma [65, 97]. The high controllability of MPI can precisely target tumor regions for localized heating and minimize collateral damage to surrounding tissues, and its non-invasive temperature monitoring capabilities further enhance its therapeutic potential [98]. Moreover, the integration of SPIONs with therapeutic agents is a compelling strategy for drug delivery across the BBB [99]. For example, the conjugation of SPIONs with doxorubicin has demonstrated efficient drug delivery to gliomas [100]. The work of Zhu et al. [101] on the quantitative monitoring of drug release using MPI further underscores the potential of this technique for precise drug delivery in brain tumor treatment.

Therefore, despite significant advances in existing technologies in tumor diagnostics [102–105], MPI still offers irreplaceable advantages compared to the existing methods. MPI locates tumors by detecting SPIONs accumulated at the tumor site and assesses the level of tumor-associated molecular content based on quantitative characteristics. It offers some advantages over MRI, such as magnetic resonance spectroscopy, diffusion weighted imaging, diffusion tensor imaging, and perfusion weighted imaging. First, MPI exhibits higher sensitivity than MRI [20, 30], which is helpful to capture weaker tumor signals at an early stage and identify tiny tumor metastases. Second, MPI shows a more linear relationship between the signal intensity and the concentration of the probes than MRI [106], and thereby, helps to assess the risk of the tumor more precisely. Additionally, compared to PET/CT, the most significant advantage of MPI is the absence of ionizing radiation, allowing for multiple repeated scanning and even real-time detection.

In the future, the exceptional sensitivity of MPI can help researchers probe deeper into the complex biological processes that drive tumor formation and progression. By conjugating SPIONs with specific biomarkers, MPI can trace the intricate pathways involved in cancer development, from the initial stages of transformation to the advanced phases of malignancy, facilitating a more comprehensive understanding of oncogenic mechanisms. Additionally, MPI may provide neurosurgeons with highly sensitive images before or during surgery to assist in the removal of brain tumors. Given the complex neural fiber connections between tumors and surrounding brain tissue, avoiding damage to critical neural fiber networks during surgery is essential to minimize negative impacts on brain function. Therefore, understanding the 3D spatial relationship between the tumor and functional areas before surgery is crucial. However, achieving this goal requires the integration of multimodal imaging. Several studies have successfully combined MPI with multiple modalities to enhance glioma visualization [26, 48, 70]. Combining MPI with these established modalities could offer clinicians a comprehensive toolset. This facilitates to integrate the anatomical, functional, and molecular information to improve surgical planning, real-time monitoring during interventions, and follow-up assessments [107]. For example, MPI combined with MRI can precisely map the 3D spatial relationships between tumors and critical neural fibers, reducing damage to vital neural networks during surgery and aiding in the precise resection of tumors [53, 108]. This multimodal approach overcomes the limitations of single-modality imaging by offering complementary insights that are crucial for early tumor detection and the precise mapping of molecular and metabolic profiles [54, 109, 110].

### Cerebrovascular diseases

The integrity of the cerebral vascular system is crucial for maintaining the delicate environmental equilibrium essential for brain health. Cerebrovascular diseases or nerve compressions may arise from pathological alterations within these vascular structures, resulting in subsequent damage to the brain’s structure and function. Over two-thirds of patients with cerebrovascular diseases can develop early neurological deterioration due to hematoma enlargement within 48 h [61, 111]. Therefore, rapid lesion localization is critical in the therapeutic management of such diseases. MPI has demonstrated high temporal resolution in various applications, enabling rapid lesion localization [31]. Another notable advantage of MPI is its ability to detect capillary blood signals with minimal interference from background tissue, providing a superior SNR at vascular sites. This characteristic renders MPI a promising tool for assessing vascular perfusion [25], a key aspect in managing neurovascular disorders. Preclinical trials have further underscored the efficacy of MPI, as exemplified by the utilization of novel multicore nanoparticles for visualizing the inferior vena cava and abdominal aorta in rodent models [22]. In a groundbreaking study, SPIONs were injected into the tail vein of rats and trapped in the lung capillaries after 10 min, accomplishing the first-ever lung perfusion imaging using MPI [112]. These findings underscore MPI’s significant potential for the diagnosis of cerebrovascular diseases and call for further research to optimize its clinical applicability.

**Fig. 6:**
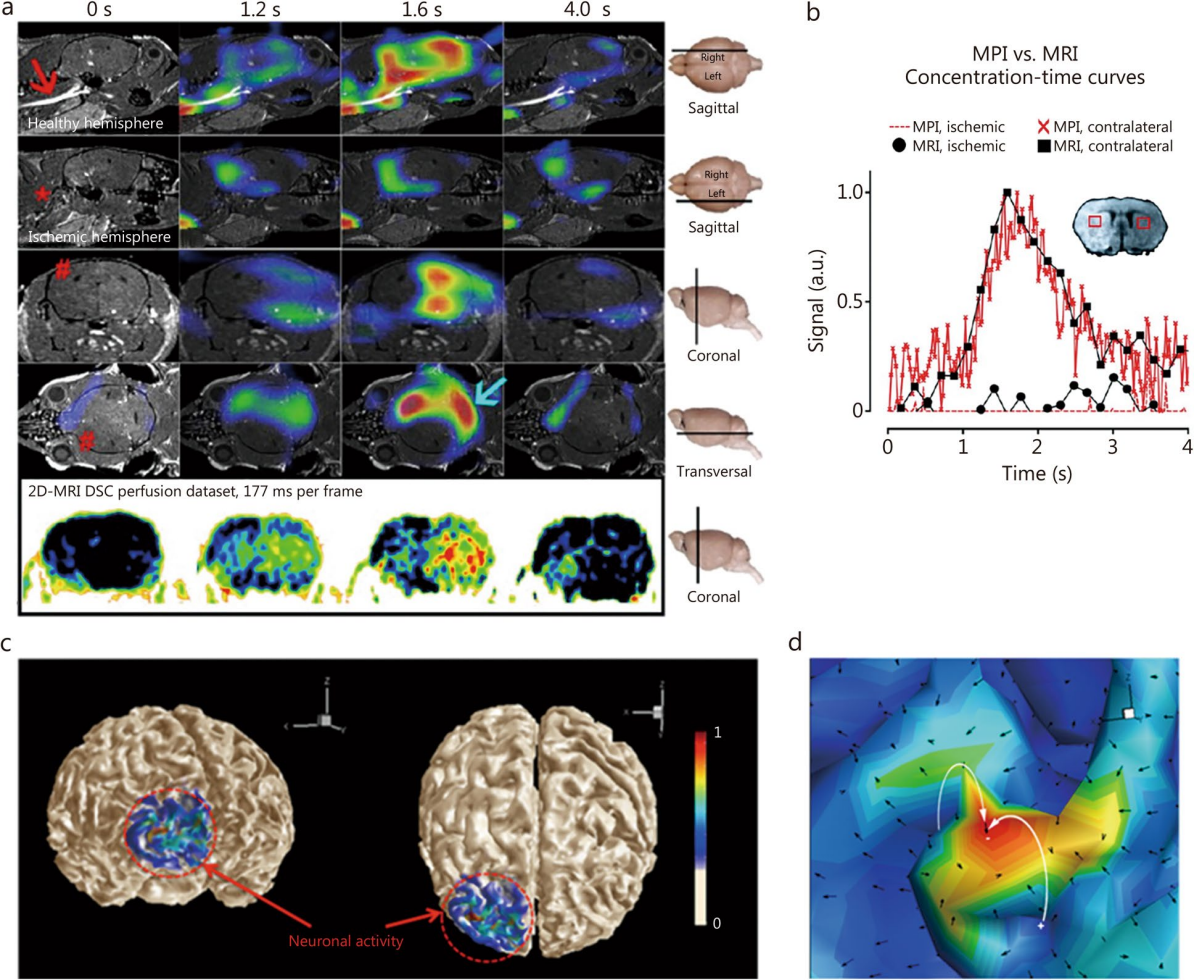
The potential of magnetic particle imaging (MPI) in cerebrovascular and neurodegenerative diseases. MPI was used to detect cerebral perfusion and stroke (**a** and **b**). **a** MPI clearly detected the ischemic area (red hash mark).** b** Concentration–time curves showed that MPI yields similar results to magnetic resonance imaging (MRI) but with higher temporal resolution. Reprinted with permission from Ref. [60] (**a** and **b**). The magnetoelectric effect of magnetoelectric nanoparticles (MENs) was proposed to simulate local nerve electrical activity (**c** and **d**). When coupled with MPI, it can be used to map the electric field activity of the brain in real time. **c** Normalized de-modulated MPI-MEN images of 2 different views of frontal lobes. **d** The insert shows a detailed normalized three-dimensional field profile in the region of firing. Reproduced with permission from Ref. [59] (**c** and **d**)

The role of MPI in the diagnosis of stroke is particularly noteworthy. Its strengths for blood flow imaging make it an invaluable tool for detecting ischemic stroke. A preclinical study investigating MPI for ischemic stroke detection [60] involved injecting mice with induced cerebral ischemia with LS-008. Compared to MRI, MPI exhibited shorter detection times and higher temporal resolution for identifying ischemic regions. Repeated tests revealed an ischemic stroke area of several cubic millimeters, validating MPI as an effective diagnostic tool for ischemic stroke (Fig. 6a, b) [60]. However, accurately estimating absolute perfusion parameters is challenging due to poor spatial resolution of MPI [71]. The development of a specialized receive coil for mouse brain improved the spatial resolution and sensitivity and achieved a spatial resolution of 0.50 mm × 0.50 mm × 0.39 mm at 46 FPS, allowing perfusion detection in small arterial vessels [68]. MPI also holds promise for evaluating stroke severity by measuring blood flow velocity for stenosis determination [113, 114] and identifying vulnerable atherosclerotic plaques, which are possible causes of stroke [115]. A preliminary research involving nonhuman primates has already shown that it is possible to detect variations in tracer concentration in cerebral perfusion, which indicates the potential of MPI for clinical application [88]. Another study explored the potential of MPI in detecting acute intracranial hemorrhages in a clinical scenario [61]. In this study, intradermal injection of collagenase disrupted the BBB, inducing intracerebral hemorrhage. Then, SPIONs were injected 30 min post-bleeding, and the active bleeding was detected within minutes. With the help of multi-contrast MPI, differentiation between fluid and clotted blood areas within the hematoma was achieved, facilitating the simultaneous imaging of bleeding and cerebral perfusion. Therefore, MPI can offer different signals for the ischemic and normal tissues, respectively, enabling rapid identification of vascular occlusion or bleeding sites, and thereby providing guidance for disease treatment. Additionally, MPI has great potential to be further used in interventional surgery for stroke. First, MPI is a non-radiation imaging technology, which allows for continuous imaging during interventional surgery [116, 117]. Second, a study has shown that MPI can serve as a lacquer marked on surgical instruments (such as balloon catheters) to achieve the tracking of these instruments during surgery [117]. These advantages make MPI a promising tool for interventional surgery for stroke [118, 119]. However, the interventional application of MPI is still in the phantom experimental stage. Further research and technological development are needed. The future envisions the integration of MPI with magnetic catheter technology, which promises to deliver more precise and effective interventional treatments.

In conclusion, MPI demonstrates significant potential in the diagnosis and interventional treatment of cerebrovascular diseases, particularly in stroke. However, due to their extended half-lives, the SPIONs utilized for MPI may potentially interfere with subsequent MRI examinations. The residual SPIONs within the body can induce local magnetic field heterogeneities, which not only reduces SNR, but also produces more serious artifacts for the subsequent MRI examinations. To avoid or alleviate the interference with urgent MRI examinations, clinicians may consider allowing an appropriate time interval between MPI and subsequent MRI. Research is underway to develop SPIONs with faster clearance rates from the body to minimize the disruptions to the subsequent imaging checks.

### Neurodegenerative diseases

Neurodegenerative disorders present a profound challenge in the field of neuroimaging. They are characterized by the relentless deterioration, and the shrinkage of brain neurons and their synaptic connections, culminating in a spectrum of cognitive and motor deficits [8]. Conditions such as AD, PD, and HD are typical examples of progressive pathologies that significantly affect millions of people worldwide.

Functional magnetic resonance imaging (fMRI) is usually used to explore the neural mechanisms of these neurodegenerative diseases. However, recent studies have demonstrated that functional magnetic particle imaging (fMPI) can provide higher contrast-to-noise ratio (CNR) compared to fMRI [57, 62], suggesting that fMPI has the potential to diagnose neurodegenerative diseases. A study utilizing a single-sided magnetic particle detector to monitor changes in cerebral blood volume (CBV) changes during hypercapnia demonstrated the higher CNR compared with fMRI [57, 58]. In a hypercapnia rat model, fMPI showed a consistent about 10% signal difference during alternated hyper/hypocapnia, exceeding about 1% difference typically reported for fMRI [57]. CBV, a critical factor for brain metabolism, has been identified as an important index for the early diagnosis and treatment of neurodegenerative diseases [120, 121]. Dynamic monitoring of CBV helps to observe the severity of neurodegenerative diseases, because the reduced brain blood volume correlates with the disease progression and severity [122]. Comparative studies between 2.83 T/m fMPI and 9.4 T/m fMRI in a hyper/hypocapnia mouse model revealed that fMPI’s average CNR for CBV was 2–6 time higher, despite its lower spatial resolution [62]. The potential of fMPI in neurodegenerative disease diagnosis is evident, with the promise of greater advantages as its gradients approach those of fMRI.

Beyond CBV monitoring, researchers are exploring the potential of MPI in measuring neuronal electrical activity, particularly in the firing of neurons in patients with neurodegenerative diseases. Real-time monitoring of local electric fields in response to neural activity could illuminate the etiological underpinnings of neurodegenerative diseases, because aberrant or absent electrical signals in neurons can result in the symptoms. Although the studies about fMPI in neurodegenerative diseases are very limited, a simulation study has explored the potential of MPI in this field [59]. In this study, when neurons emit electrical signals, electric fields are generated in local areas, and then the magnetoelectric nanoparticles (MENs) can sense the changes in the electric fields (Fig. 6c, d) [59]. The electric fields affect the magnetization state of MENs through magnetoelectric effects, and this magnetization alteration can be further detected by the MPI device. Theoretically, if MENs can be designed in the future, MPI will be capable of measuring neural electrical activity. Compared to magnetoencephalography (MEG), which is commonly used to measure the electromagnetic physiological signals of the brain [123], MPI has a higher spatial resolution. Specifically, for MPI, when the FFR moves within the region containing SPIONs in the imaging space, these particles will generate a magnetization response, which induces a voltage signal in the receiving coil. The spatial location of the signal source can be directly determined from the MPI image. This capability of direct spatial localization gives MPI an advantage in imaging accuracy, particularly when monitoring neuronal activity. In contrast, MEG detects the subtle magnetic field fluctuations generated by neural activity in the brain. The distribution of these magnetic field signals on the scalp is a complex superposition of multiple intracerebral sources. Deciphering the location and activity intensity of intracerebral sources from the scalp’s magnetic field distribution necessitates solving a complex inverse problem [124, 125], which is mathematically challenging. The superposition of signals from multiple sources further amplifies the difficulty of the source localization in MEG. Consequently, MPI has the potential to offer a more accurate and direct approach for visualizing neuronal electrical signals.

### Other brain diseases

Besides the neurological diseases mentioned above, MPI has shown potential in other brain diseases, particularly multiple sclerosis (MS). MS is an immune-mediated inflammatory demyelinating disease of central nervous system [126]. One of its early pathological features is inflammation in the brain and spinal cord [127]. MPI technology, utilizing SPIONs as tracer agents, has been shown to be highly sensitive to inflammatory sites and thereby precisely monitor inflammatory states [115, 128, 129], providing a possible new method for the diagnosis of MS. Chandrasekharan et al. [129] first used MPI to trace leukocytes in inflammation and infection sites. In this study, they designed Ly6G-SPIONs that specifically target the Ly6G antigen on neutrophils, and successfully detected myositis inflammation with high sensitivity. Tong et al. [115] developed a multimodal nanoprobe 5HFeC that specifically targeted myeloperoxidase (MPO) and recognized atherosclerotic plaques in vivo. Thus, 5HFeC showed a high sensitivity response to MPO and therefore was able to successfully detect the activity of MPO and assess the severity of plaques in atherosclerotic mouse models. Gao et al. [128] constructed an MPI probe ESPVPN, which effectively targeted vascular cellular adhesion molecule-1 in inflammation sites of lung injury, achieving a visual assessment of inflammation levels by monitoring the changes of this molecule. With the advantages of high sensitivity and quantification in detecting inflammatory diseases, MPI is anticipated to be used in the diagnosis of brain diseases such as MS. First, MPI can identify MS-related inflammatory markers or specific molecules associated with myelin sheath destruction (such as immune cells) through targeted modification of SPIONs, providing support for exploring the disease mechanisms. Second, due to the advantages of MPI in cell tracking, MPI may further be used to monitor stem cell therapy in MS, tracking the location and survival of these cells in the brain, providing real-time feedback for neural regeneration.

## Multi-contrast and human-sized MPI strategies

To advance the clinical translation of MPI, researchers are focusing on two key areas: the development of multi-contrast MPI and the construction of MPI devices suitable for human-scale imaging [15]. Advances in multi-contrast MPI are set to expand the clinical application scope of MPI, particularly in the diagnosis of tumors and vascular diseases [35]. By facilitating the non-invasive and in vivo acquisition of biomolecular activity data during disease progression, these advancements promise to deepen our understanding of the pathogenesis and progression of various diseases. Moreover, the ongoing development and evaluation of human-scale MPI devices are anticipated to accelerate the transition of this technology from research to clinical practice.

### Multi-contrast MPI

The scientific community is actively exploring the applications of multi-contrast MPI, which are anticipated to become a mainstay in the field’s future. Within the field of MPI, different SPIONs exhibit distinct binding characteristics with cells in living organisms. Notably, even identical SPIONs can exhibit tissue-specific distribution patterns to the different tissues that differentiate diseased from healthy regions. This diversity in the behavior of SPIONs allows for the simultaneous visualization of multiple SPIONs in a single imaging session, thereby offering insights into the distinctive properties of different tissues. For example, Rahmer et al. [130] utilized harmonic response signals to differentiate between the fluid and powder forms of SPIONs. The relaxation time constant of SPIONs, derived from their MPI signal, has been effectively employed to classify various commercially available SPIONs, including Perimag, Nanomag-MIP, and VivoTrax [131]. Additionally, in vivo imaging of SPIONs of two distinct sizes—12 nm and 20 nm—has been achieved by assigning them distinct colors based on their point spread function [132]. In this study, after intravenous administration in mice, these particles exhibited distinct biodistribution patterns, with the 12 nm and 20 nm SPIONs accumulating in the liver and lung, respectively.

These distinctive features of multi-parametric imaging open new avenues for studying brain tumor diseases. By analyzing critical parameters such as the concentration, targeting specificity, viscosity, and temperature of SPIONs, we can precisely capture molecular information about tumors, including the quantity, type, tissue characteristics, and energy metabolism of molecules [34]. For example, within the tumor microenvironment, antibody-modified SPIONs can specifically target the receptors on the surface of tumor cells, revealing the interactions between the tumor cells and surrounding tissues. Unmodified SPIONs, on the other hand, can infiltrate the interstitial fluid of tissues, providing vital reference information for tumor diagnosis through their distribution patterns in different tissues and lesion areas. Additionally, the research by Utkur et al. [133] has highlighted the potential of relaxation-based color MPI to monitor changes in viscosity. They established a correlation between the relaxation time constants and the viscosity levels, thereby enabling viscosity mapping. This heightened sensitivity to the intracellular viscosity positions MPI as a potentially valuable diagnostic tool for diseases like cancers and atherosclerosis, for which increased intracellular viscosity is a characteristic indicator.

For the cerebrovascular diseases, MPI not only can display different blood states based on the parameters of SPIONs for disease diagnosis and severity assessment, but also has the potential to revolutionize disease understanding through innovative approaches. A groundbreaking study conducted in 2019 developed a model for MPI-guided endovascular stent implantation [134]. Researchers utilized an MPI-visible paint based on SPIONs to label guide wires and fill blood vessels with a 10 mmol Fe/L SPIONs solution [116, 117]. This pioneering method allowed for the visualization of the entire process of balloon angioplasty and stent placement, suggesting that MPI could emerge as a novel vascular interventional technique, which is termed as MPI angiography or MPA. This technique could serve as a radiation-free adjunct to other imaging modalities, offering a significant advantage in the field of vascular interventions.

### Human-sized MPI

**Fig. 7:**
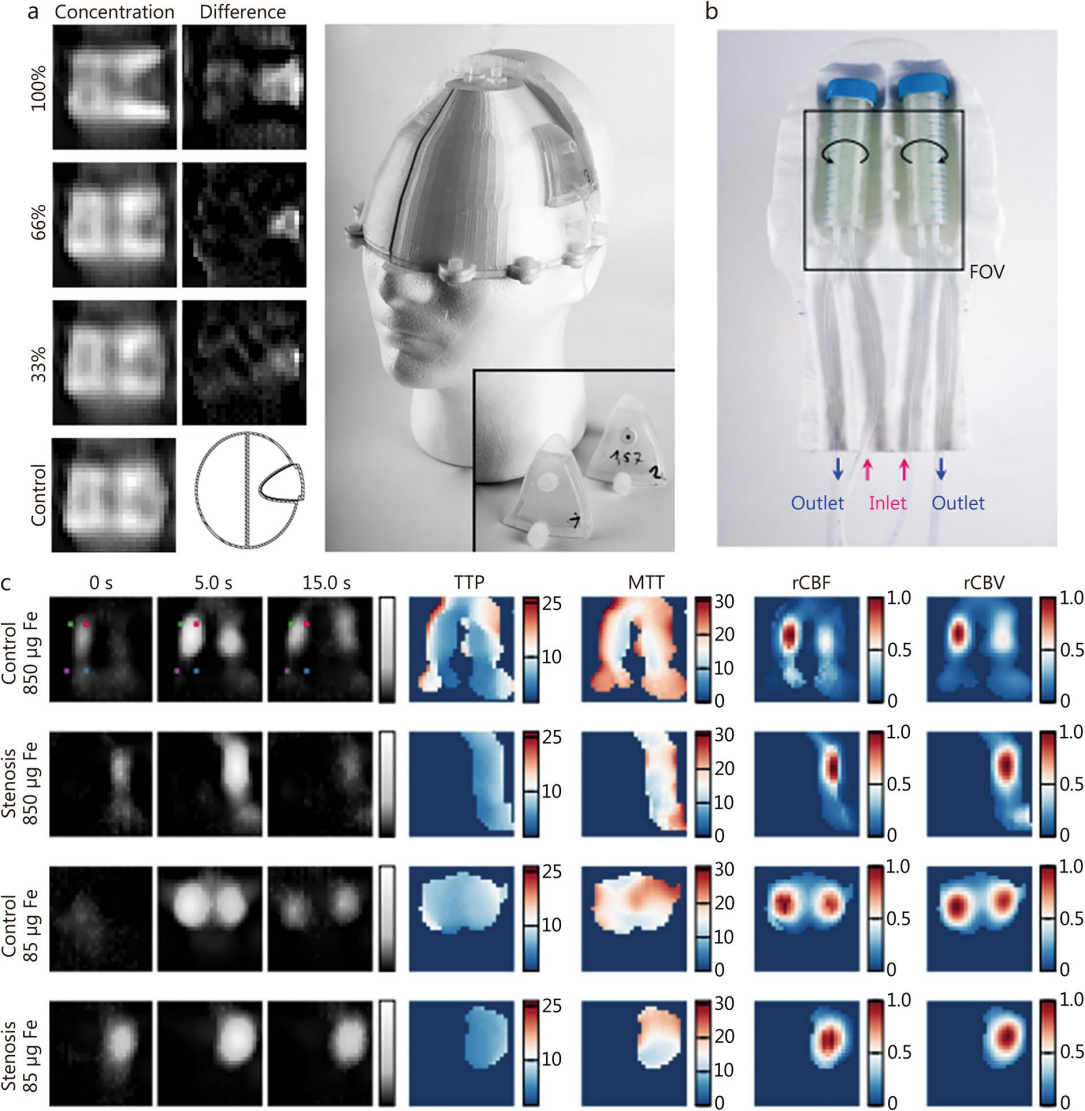
The human-size magnetic particle imaging (MPI) for stroke. **a** In static stroke experiments, concentration differences can be observed in the stroke parts compared to control measurements. **b** The complete phantom, consisting of two tubes filled with glass spheres, was used to simulate blood flow in vessels. **c** Dynamic imaging results of the brain imager, including time to peak (TTP), mean transit time (MTT), relative cerebral blood flow (rCBF) and relative cerebral blood volume (rCBV). Reprinted with permission from Ref. [56] (**a**–**c**). FOV field of view

The current landscape of MPI systems is predominantly geared towards preclinical use. Its clinical translation is limited by the aperture size, and therefore needs specially designed MPI devices that can accommodate larger and more complex human anatomy. Notable progress has been made by researchers like Mason et al. [135], who designed MPI systems with clinical utility in mind. Companies such as Magnetic Insight Inc. are also leading initiatives to develop systems suitable for clinical use. The earliest prototype appeared in 2019 [56], marking a significant milestone in the development of human-scale MPI. This prototype was designed for continuous bedside monitoring of patients with strokes in intensive care units (ICU) (Fig. 7) [56], achieving a sensitivity limit of 14.7 ng Fe/ml at a frame rate of 2 Hz and a spatial resolution of 5 mm under a gradient strength of only 0.2 Tm^−1^u_0_^−1^. The latest iteration from the same team, which included the use of a superconductor, has further refined these capabilities [63]. This superconducting MPI system employed a low frequency focusing field to scan the entire imaging aperture by the partial FOV method, achieving a spatial resolution of less than 5 mm and a frame rate of 4 Hz at the gradient of 2.5 Tm^−1^u_0_^−1^ [67]. One of the most compelling advantages of MPI in the clinical setting is its potential for continuous bedside monitoring, which is currently unparalleled by other imaging modalities. MPI can provide a mobile, non-invasive, and real-time imaging technique for monitoring brain perfusion within the ICU, potentially revolutionizing the care of patients with vascular diseases such as stroke. Furthermore, there is ongoing work towards developing large-scale whole-body MPI scanners. For instance, a clinical-scale remote magnetic braking system has been established to facilitate image-guided remote magnetic control, demonstrating real-time imaging capabilities with a spatial resolution of 4 mm in the X-direction and 2 mm in the Z-direction in raw pork [55]. Such large-scale MPI models, integrated with remote magnetic control, are poised for future targeted invasive brain medical procedures [55, 136].

For the transition from preclinical to clinical use, the design and development of MPI systems must prioritize patient safety and address potential adverse effects of time-varying magnetic fields, such as tissue heating, eddy currents, peripheral nerve stimulation (PNS), and specific absorption rate (SAR) issues [137, 138]. Encouragingly, a research indicates that the magnetic field characteristics used in body scanning are within safe limits for PNS and SAR, provided that the device is operated within specific frequency ranges [137]. Additionally, key performance metrics also need to be considered. For clinical use, MPI systems need to achieve picomolar sensitivity, sub-millimeter spatial resolution, at least 5 FPS temporal resolution, and less than 100 ms real-time latency [67]. These enhancements will be crucial for MPI to meet the demands of clinical practice and provide effective diagnostic and monitoring capabilities for a wide range of neurological conditions.

## Conclusions and prospects

Although the applications of MPI are now mainly focused on the small animal imaging, these feasibility studies still demonstrate the great potential of MPI in the future diagnosis of human brain diseases. Compared with traditional imaging techniques, MPI offers notable advantages, including high sensitivity, high temporal resolution, high controllability, and the absence of radiation exposure.

The most important advantage of MPI is the exceptionally high sensitivity for the detection of very low concentration of SPIONs [20]. Thus, compared with other imaging methods, MPI exhibits great advantages in the early identification of brain pathologies, the precise localization of minute lesions, and the surveillance of metastatic dissemination. For the diagnosis of brain tumors, MPI may precisely evaluate the tumor microenvironment and angiogenesis at early stages, which is vital for timely tumor detection. For surgical resection of brain tumors, MPI aids in the identification of residual lesions and the detection of metastatic deposits. Furthermore, for the cerebrovascular diseases, MPI is expected to detect smaller bleeding points on the microvessels, contributing to ensure timely intervention of patients before the lesions expand.

The high temporal resolution of MPI allows for rapid image acquisition [31, 60], enabling real-time monitoring of cerebral blood flow and neural activity. In the management of cerebrovascular diseases, MPI is expected to swiftly pinpoint vascular occlusions or bleeding sites [60], providing a critical time window for emergency intervention. In the perioperative period of neurosurgery, MPI may monitor cerebral blood flow changes in real-time, assisting clinicians in evaluating cerebral perfusion states and preventing intraoperative ischemia [56, 71]. Its utility extends to the hemodynamic assessment of conditions like aneurysms and arteriovenous malformations, where continuous evaluation of blood flow and vascular wall stress can offer timely alerts, aiding physicians in assessing the risk of aneurysm rupture.

The high controllability of MPI through the precise manipulation of the FFR in the imaging space, allows for the exact control of SPIONs distribution, offering new methods for precise magnetic hyperthermia and targeted drug delivery. In brain tumor treatment, MPI may accurately control the heating area [97], achieving localized tumor therapy while protecting surrounding healthy tissue [65]. Coupled with the quantifiable imaging and targeted probes, this enables the quantitative release of drugs within the body [101], paving the way for personalized medicine.

As a radiation-free imaging technology, MPI can safely and harmlessly perform long-term, repeated, and multiple imaging sessions. This reduces the risk of radiation exposure for medical staff and patients, providing a safe option for the treatment where continuous imaging is necessary (e.g., in interventional surgeries). In brain disease management, such as ICU monitoring, radiochemotherapy surveillance, and patient follow-ups, MPI is expected to serve as a bedside device for continuous medical surveillance, offering uninterrupted patient care.

Despite significant advancements in preclinical research, there are still many challenges in its translation from animal experiments to human clinical applications. The majority of MPI devices are currently tailored for small animal research with limited bore diameters, restricting the application in human patients. Scaling up MPI bore to accommodate human-scale imaging presents technical challenges, including an increase in power consumption and a reduction in sensitivity due to the weakened coupling between the receiver coils and the magnetization of the tracers. Enlarging the MPI device also leads to increased physiological stimulation and tissue heating, which are unacceptable for clinical applications. Additionally, in the interest of human safety, the employment of low concentrations of SPIONs may become necessary in clinical scenarios, which could decrease signal strength and image quality. Therefore, it needs the development of new hardware technologies and image reconstruction strategies to increase device size without compromising patient safety and minimize device power consumption without reducing imaging performance, such as resolution, sensitivity, and SNR. A recent study reported that a superconducting MPI device exhibits potential for human-scale imaging [63]. However, the temporal resolution and spatial resolution have not yet met the desired standards, indicating that further technological innovation and optimization in device design are required.

The biocompatibility and safety of SPIONs are also a critical issue, particularly in the upcoming brain disease studies. Although the metabolic pathways of SPIONs in small animal models are relatively well understood [44], the long-term metabolism, tissue distribution uniformity, and biodegradability in human still require further investigation. Large animal experiments, which more accurately mimic the complex physiological environment of humans, are essential for validating the long-term safety and biocompatibility of SPIONs. These studies using large animals can also facilitate determining the penetration depth of SPIONs, the circulation half-life in brain tissues, the complete clearance time in the body, and the influence of cerebral blood flow variations on imaging quality. Additionally, for studies of brain diseases, the design of SPIONs must be customized to align with the pathological nuances of various brain diseases. For instance, for brain tumor detection, SPIONs should exhibit high affinity and retention capabilities to accurately target tumor cells. For cerebrovascular diseases, SPIONs should be designed to have a brief half-life to avoid or alleviate the interference with MRI that might be performed after MPI. Moreover, for the early detection of neurodegenerative diseases, SPIONs should demonstrate adequate sensitivity and selectivity to identify very small foci of neural damage.

In conclusion, MPI exhibits great potential in the diagnosis and treatment of brain tumors, cerebrovascular diseases, and neurodegenerative diseases due to its multiple advantages. However, for its widespread clinical application, further research is needed in several areas, including the advancement of equipment, the improvement of imaging algorithms, and the optimization of tracers. With these enhancements, MPI will become a routine tool in neurology, providing more effective solutions for early diagnosis and precise treatment of neurological diseases.

## Data Availability

No data was used for the research described in the article.

## Abbreviations

AD: Alzheimer’s disease
AMF: Alternating magnetic field
BBB: Blood-brain barrier
CBV: Cerebral blood volume
CNR: Contrast-to-noise ratio
CT: Computed tomography
FFL: Field-free line
FFP: Field-free point
FFR: Field-free region
fMRI: Functional magnetic resonance imaging
fMPI: Functional magnetic particle imaging
FOV: Field of view
FPS: Frames per second
HD: Huntington’s disease
ICU: Intensive care unit
MEG: Magnetoencephalography
MENs: Magnetoelectric nanoparticles
MPI: Magnetic particle imaging
MPO: Myeloperoxidase
MRI: Magnetic resonance imaging
MS: Multiple sclerosis
NPCs: Neural progenitor cells
PET: Positron emission tomography
PD: Parkinson’s disease
PNS: Peripheral nerve stimulation
SAR: Specific absorption rate
SNR: Signal-to-noise ratio
SPIONs: Superparamagnetic iron oxide nanoparticles
3D: Three-dimensional
